# Usefulness of sputum gram stain for etiologic diagnosis in community-acquired pneumonia: a systematic review and meta-analysis

**DOI:** 10.1186/s12879-019-4048-6

**Published:** 2019-05-10

**Authors:** Gaspar Del Rio-Pertuz, Juan F. Gutiérrez, Abel J. Triana, Jorge L. Molinares, Andrea B. Robledo-Solano, José L. Meza, Orlando M. Ariza-Bolívar, Jorge Acosta-Reyes, Argenis Garavito, Diego Viasus, Jordi Carratalà

**Affiliations:** 1 Faculty of Medicine, Division of Health Sciences, Hospital Universidad del Norte and Universidad del Norte, Barranquilla, Colombia; 2 Faculty of Public Health, Division of Health Sciences, Universidad del Norte, Barranquilla, Colombia; 3 Clínica Medilaser S.A. - Sucursal Florencia, Fundación Universitaria Navarra, Florencia, Colombia; 4 Infectious Disease Department, Hospital Universitari de Bellvitge and Institut d’Investigació Biomèdica de Bellvitge (IDIBELL), Barcelona, Spain; 5 Faculty of Medicine, Clinical Sciences Department, University of Barcelona, Barcelona, Spain; 6 Spanish Network for Research in Infectious Diseases (REIPI RD16/0016/0005), Instituto de Salud Carlos III, Madrid, Spain

**Keywords:** Community-acquired pneumonia, Sputum, Gram, Sensitivity, Specificity, Meta-analysis

## Abstract

**Background:**

Implementation of sputum Gram stain in the initial assessment of community-acquired pneumonia (CAP) patients is still controversial. We performed a systematic review and meta-analysis to investigate the usefulness of sputum Gram stain for defining the etiologic diagnosis of CAP in adult patients.

**Methods:**

We systematically searched the Medline, Embase, Science Direct, Scopus and LILACS databases for full-text articles. Relevant studies were reviewed by at least three investigators who extracted the data, pooled them using a random effects model, and carried out quality assessment. For each bacterium (*Streptococcus pneumoniae*, *Haemophilus influenzae*, *Staphylococcus aureus*, and Gram-negative bacilli), pooled sensitivity, specificity, positive and negative likelihood ratios were reported.

**Results:**

After a review of 3539 abstracts, 20 articles were included in the present meta-analysis. The studies included yielded 5619 patients with CAP. Pooled sensitivity and pooled specificity of sputum Gram stain were 0.59 (95% CI, 0.56–0.62) and 0.87 (95% CI, 0.86–0.89) respectively for *S. pneumoniae*, 0.78 (95% CI, 0.72–0.84) and 0.96 (95% CI, 0.94–0.97) for *H. influenzae*, 0.72 (95% CI, 0.53–0.87) and 0.97 (95% CI, 0.95–0.99) for *S. aureus*, and 0.64 (95% CI, 0.49–0.77) and 0.99 (95% CI, 0.97–0.99) for Gram-negative bacilli.

**Conclusion:**

Sputum Gram stain test is sensitive and highly specific for identifying the main causative pathogens in adult patients with CAP.

**Trial registration:**

This study has been registered at PROSPERO International prospective register of systematic reviews under registration no. CRD42015015337.

**Electronic supplementary material:**

The online version of this article (10.1186/s12879-019-4048-6) contains supplementary material, which is available to authorized users.

## Background

Community-acquired pneumonia (CAP) is a frequent infection with significant morbidity and mortality, especially in extreme ages of life and in patients who have underlying diseases [1, 2]. CAP is also related with a high economic burden, and considerable long-term effects on quality of life and prognosis [3, 4].

Several tests are recommended to establish the causative pathogen of CAP. Although the sputum Gram stain is a rapid, simple and low-cost method, its role in the initial assessment of patients with CAP is still controversial. Studies have raised doubts about its utility due to the difficulty in obtaining the sample and its limited sensitivity, and overall impact on decision-making [5], but other authors favor its use [6]. The identification of the causative pathogen in CAP by sputum Gram stain may facilitate the use of a targeted antimicrobial therapy, thus decreasing the collateral damage (selection of drug-resistant pathogens and the development of colonization or infection with multidrug-resistant organisms), saving costs and limiting the risk of adverse reactions.

The current guidelines for the management of CAP contain a variety of recommendations for performing sputum Gram stain [2, 7, 8]. While some of them advocate its routine use, others recommend it in moderate to severe cases, in circumstances in which it can be processed at the place where it was taken, or when it would alter empirical therapy. Up to now, only one meta-analysis has evaluated the accuracy of sputum Gram stain for determining the causative pathogens of CAP [9], and in fact that study focused only on pneumococcal pneumonia. What is more, that meta-analysis was carried out nearly two decades ago, and since then several studies analyzing the usefulness of sputum Gram stain in CAP have been published.

We performed a systematic review and meta-analysis to investigate the usefulness of sputum Gram stain for determining the etiologic diagnosis of CAP in adult patients.

## Methods

The present systematic review and meta-analysis was performed following the guidelines for meta-analyses of observational studies in epidemiology (MOOSE) [10]. We systematically searched the Medline, Embase, Science Direct, Scopus and LILACS (Literatura Latino-Americana e do Caribe de Informação em Ciências da Saúde) databases for full-text articles that assessed the accuracy of sputum Gram stain for determining the etiology of CAP in adults. The search strategy included the terms “community-acquired pneumonia”, “sputum” and “Gram”. We searched in the databases from their inception to November, 2014. Two investigators independently conducted the literature search.

To be eligible, the studies should have a standard definition for CAP diagnosis, which was defined as the evidence of a pulmonary infiltrate on chest radiography plus at least one of the following: sputum production, fever or hypothermia, cough, pleuritic chest pain, or leukocytosis or leukopenia. Additionally, the sputum Gram stains had to be compared with an independent gold standard. Studies were rejected if they were performed in immunosuppressed patients or in patients under 16 years of age. Patients were considered immunosuppressed if they have neutropenia, transplantation or splenectomy, HIV, immunoglobulin deficiencies, and those who were receiving chronic corticosteroid therapy or other immunosuppressant therapies.

The studies had to provide sufficient information for the creation of a 2 × 2 diagnostic table. Only publications written in English and Spanish were included. We excluded studies that evaluated animal models, editorials, letters, reviews, abstracts from congresses and case reports.

### Procedures

We excluded non-relevant studies by the review of the abstracts. At least three investigators reviewed potentially relevant studies. They also carried out data extraction and assessed the quality of studies in a blinded manner. Quality Assessment of Diagnostic Accuracy Studies (QUADAS-2) were used to evaluate the methodology quality of each study included [11]. Differences about quality assessment or eligibility of studies were resolved by consensus. The main features of the studies included, such as author, year and journal of publication, country and type of study design, sputum quality, sputum Gram stain results, sensitivity and specificity of sputum Gram stain to predict the causative pathogen of CAP (*S. pneumoniae*, *H. influenzae*, *S. aureus*, and Gram-negative pathogens), and sample size were extracted using a standardized form.

### Statistical analysis

We extracted or estimated values for true positives, true negatives, false negatives and false positives for each included study and calculated sensitivity and specificity with 95% confidence intervals (95% CI). For each causative pathogen (*S. pneumoniae*, *H. influenzae*, *S. aureus*, and Gram-negatives such as *K. pneumoniae* and *P. aeruginosa*), pooled sensitivity, specificity, positive and negative likelihood ratios (LR) were reported. It has been proposed that a positive LR > 10 or a negative LR < 0.1 is likely to identify a clinically useful test [12]. In addition, we plotted receiver operating characteristic curves (ROC) and calculated the area under the curve (AUC) with 95% CIs. We constructed a summary ROC curve for describing the relationship between sensitivity and specificity across the studies included. Heterogeneity was evaluated by Cochrane Q test and I^2^ statistics; then, we estimated the effect size by means of fixed or random models for homogeneous or heterogeneous studies respectively. The AUCs were compared as described by Hanley and MacNeil [13]. Subgroup analyses were performed in the studies which reported “good quality sputum”, “positive sputum Gram stain” and in which patients had not received previous antibiotic therapy. We included in the subgroup analysis of “good quality” sputum those studies that used the criterion of > 25 neutrophils in a × 100 microscopic field and few squamous epithelial cells (< 10 in a × 100 microscopic field). We included in the subgroup analysis of “positive sputum Gram stain” those studies that used the criterion of the presence of > 50% microorganisms of the same morphotype. For assessment of publication bias, Begg funnel plot and Egger tests were performed. The Stata software was used and the results were considered statistically significant when *p* < 0.05.

## Results

**Fig. 1:**
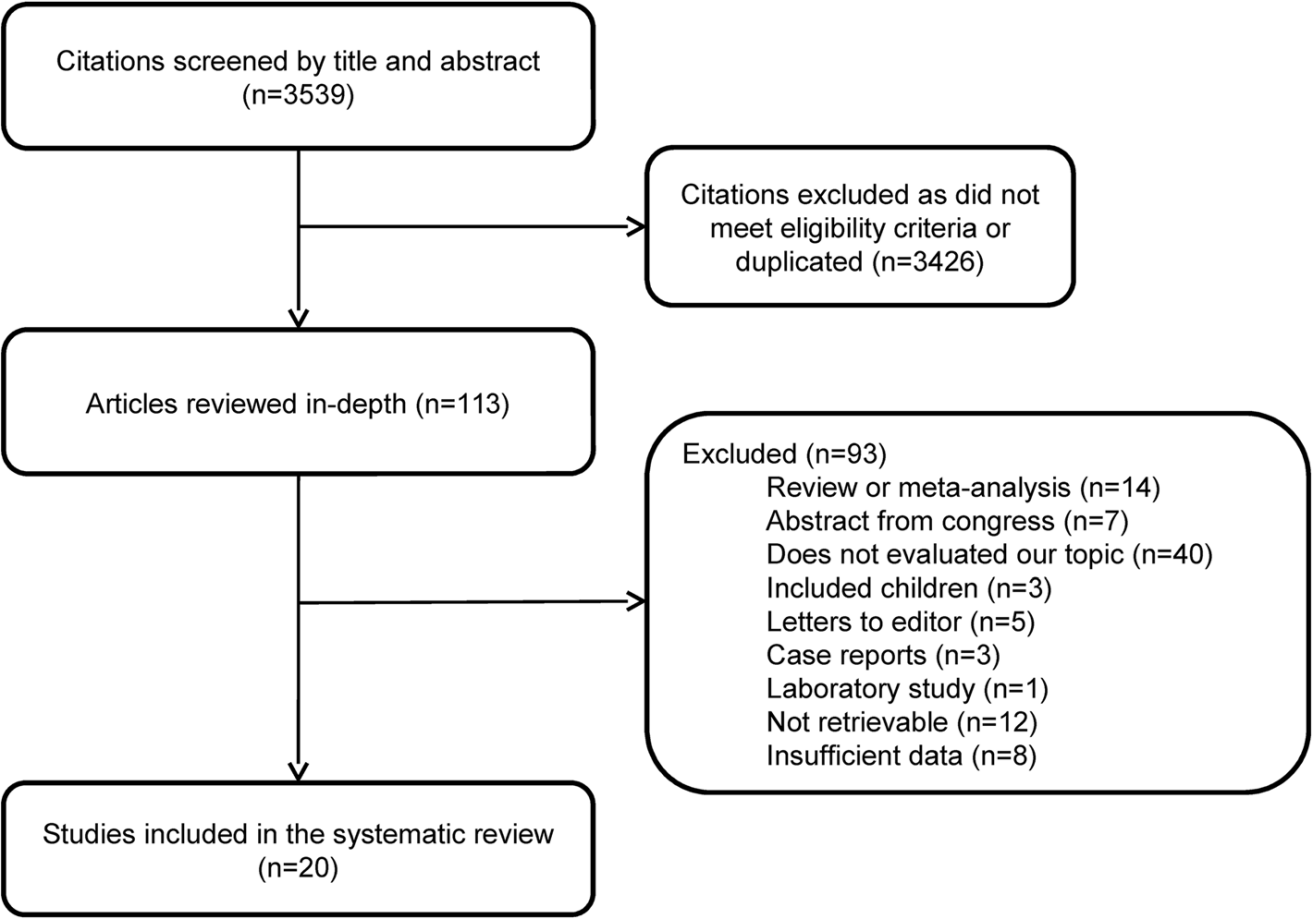
Flow chart of the literature review

After reviewing 3539 abstracts, 113 articles were considered potentially eligible and evaluated in depth. After full-text review, a further 93 articles were rejected and 20 were included in the present meta-analysis. Figure 1 summarizes the literature review process. All studies included had an observational design. Table 1 shows the main characteristics of these studies [5, 6, 14–31]. The overall methodological quality of the studies included was generally good, as measured by QUADAS-2 scores (Additional file 1: Table S1). The studies included yielded 5619 patients with CAP (range 16–1390). The number of sputum Gram stains analyzed in the studies varied from 16 to 404. Most studies used sputum culture or a combination of microbiological procedures (mainly sputum and blood cultures and urinary antigen test) as gold standard to compare the sputum Gram stains. The sensitivity and specificity of sputum Gram stain for determining CAP etiology ranged from 0 to 100% (mean of 65.7 and 84.9%, respectively). Most studies had a definition for evaluating the quality of the sputum sample and the positivity of the Gram stain, although the definitions varied from study to study. The reported mortality rates varied from 5 to 15%.

**Table 1:** Characteristics of Studies Included in the Meta-Analysis

Author	Year	Journal	Patients	Definition of positive Gram stain	Definition of good quality sample	Number of samples	Microorganism	Sensitivity (95% CI)	Specificity (95% CI)
Merrill, et al. [14]	1973	N Engl J Med	27	Unknown	Unknown	53	*S. pneumoniae*	96 (81–100)	12 (2–30)
						30	*S. pneumoniae*	43 (18–71)	88 (62–98)
Thorsteinsson, et al. [15]	1975	JAMA	16	Unknown	Unknown	16	*S. pneumoniae*	100 (75–100)	67 (9–99)
							*S. pneumoniae*	100 (75–100)	67 (9–99)
							*S. pneumoniae*	100 (75–100)	67 (9–99)
							*H. influenzae*	100 (40–100)	100 (74–100)
Rein, et al. [16]	1978	JAMA	42	Preponderant flora or > 10/oif	At least 10 PMN leukocytes	42	*S. pneumoniae*	62 (42–79)	85 (55–98)
Boerner, et al. [17]	1982	JAMA	89	> 50% of the same morphotype	PMN leukocytes in excess of epithelial cell	76	*S. pneumoniae*	62 (42–79)	85 (55–98)
Dans, et al. [18]	1984	Arch Intern Med	241	Unknown	Used a ratio of PMN leukocytes:epithelial cells	154	*S. pneumoniae*	52 (40–65)	88 (79–94)
						147	*S. pneumoniae*	63 (48–76)	80 (71–88)
BTS. [20]	1987	QJM	511	> 50% of the same morphotype	Unknown	404	*S. pneumoniae*	15 (9–22)	98 (96–99)
Lentino, et al. [19]	1987	J Clin Microbiol	249	> 50% of the same morphotype	> 25 leukocytes and < 10 epithelial cells	40	*S. pneumoniae*	56 (31–78)	95 (77–100)
Gleckman, et al. [21]	1988	J Clin Microbiol	144	> 10/oif of the same morphotype	> 25 leukocytes and < 10 epithelial cells	59	*S. pneumoniae*	69 (52–84)	83 (61–95)
Xiaoping, et al. [22]	1988	Med Microbiol Inmmunol	105	> 10/oif of the same morphotype	> 25 leukocytes and < 10 epithelial cells	95	*S. pneumoniae*	88 (62–98)	85 (75–92)
Lim, et al. [23]	1989	Med J Aust	106	> 50% of the same morphotype or > 10/oif	Unknown	40	*S. pneumoniae*	68 (48–84)	100 (74–100)
Fine, et al. [24]	1991	J Gen Intern Med	170	> 50% of the same morphotype	> 25 leukocytes and < 10 epithelial cells	36	*S. pneumoniae*	86 (42–100)	72 (53–87)
Belliveau, et al. [25]	1993	Pharmacotherapy	224	Unknown	Number of neutrophils and epithelial cells	319	*H. influenzae*	88 (68–97)	99 (97–100)
Bohte, et al. [26]	1996	Eur J Clin Microbiol Infect Dis	268	Unknown	> 25 leukocytes and < 10 epithelial cells	268	*S. pneumoniae*	65 (53–75)	76 (69–82)
Rosón, et al. [27]	2000	Clin Infect Dis	533	> 75% of the same morphotype	> 25 leukocytes and < 10 epithelial cells	210	*S. pneumoniae*	57 (46–68)	97 (92–99)
							*H. influenzae*	82 (65–93)	100 (97–100)
Ewig, et al. [5]	2001	Chest	116	Predominant morphotype	Used a score	23	*S. pneumoniae*	50 (7–93)	84 (60–97)
							*H. influenzae*	0 (0–60)	0 (0–18)
							*S. aureus*	50 (1–99)	81 (58–95)
Musher, et al. [28]	2004	Clin Infect Dis	105	Unknown	10 leukocytes for each epithelial cell	105	*S. pneumoniae*	31 (23–41)	NA
Miyashita, et al. [29]	2008	Med Sci Monit	347	> 50% of the same morphotype	> 25 leukocytes and < 10 epithelial cells	124	*S. pneumoniae*	68 (52–82)	94 (86–98)
							*H. influenzae*	78 (52–94)	100 (97–100)
Anevlavis, et al. [30]	2009	J Infect	1390	> 50% of the same morphotype	Sum of two scores > 1	178	*S. pneumoniae*	82 (72–89)	93 (85–97)
							*H. influenzae*	79 (59–92)	96 (91–99)
							Gram negative	78 (60–91)	95 (90–98)
							*S. aureus*	76 (55–91)	96 (92–99)
Ferré, et al. [31]	2011	Emergencias	608	> 75% of the same morphotype	> 25 leukocytes and < 10 epithelial cells	294	*S. pneumoniae*	47 (39–56)	94 (89–97)
						169	*H. influenzae*	73 (52–88)	95 (90–98)
Fukuyama, et al. [6]	2014	BMC Infect Dis	328	> 10/oif of the same morphotype	< 10/oif epithelial cells and > 10/oif PMN	218	*S. pneumoniae*	66 (52–78)	89 (84–94)
							*H. influenzae*	78 (64–88)	95 (90–97)
							*K. pneumoniae*	50 (12–88)	100 (97–100)
							*P. aeruginosa*	22 (3–60)	100 (98–100)
							*S. aureus*	50 (1–99)	100 (98–100)

Abbreviations: *oif* oil immersion field, *PNM* polymorphonuclear

### *Streptococcus pneumoniae*

**Fig. 2:**
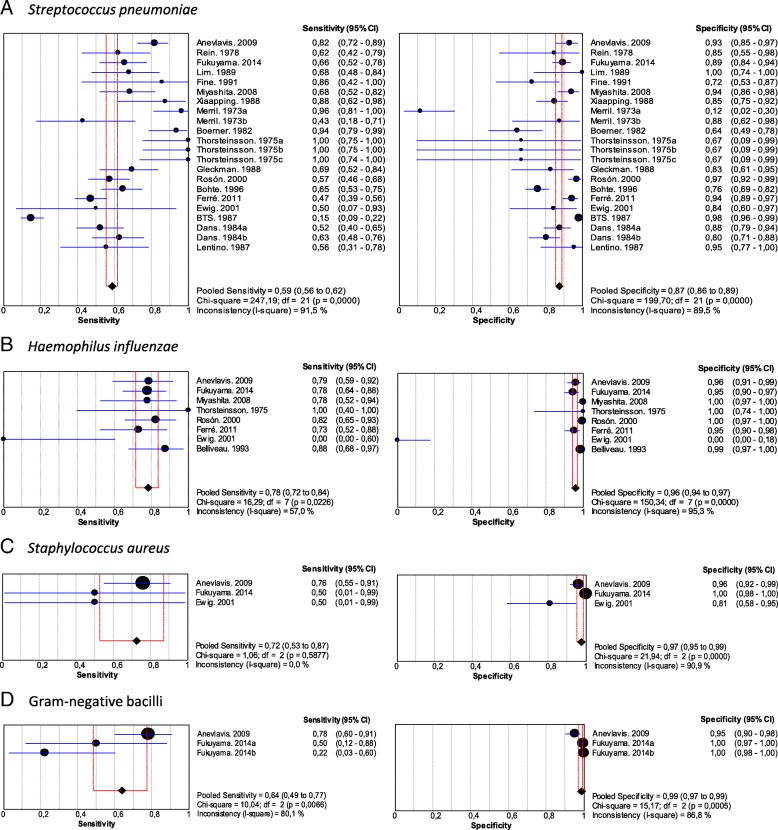
Pooled sensitivity and specificity of sputum Gram stain in community-acquired pneumonia. **a**. *Streptococcus pneumoniae*, **b**. *Haemophilus influenzae*, **c**. *Staphylococcus aureus*, **d**. Gram-negative bacilli

**Fig. 3:**
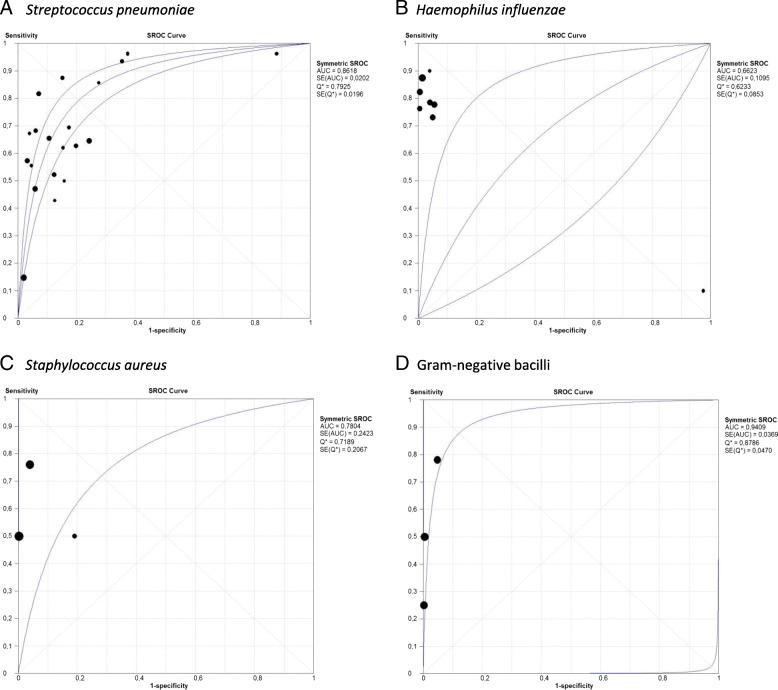
Summary area under the receiver operator characteristic curves of sputum Gram stain in community acquired pneumonia. **a**. *Streptococcus pneumoniae*, **b**. *Haemophilus influenzae*, **c**. *Staphylococcus aureus*, **d**. Gram-negative bacilli

Nineteen studies of 5395 patients with community-acquired pneumococcal pneumonia were included in this analysis (Additional file 1: Table S2). Pooled sensitivity was 0.59 (95% CI, 0.56–0.62) and pooled specificity was 0.87 (95% CI, 0.86–0.89) (Fig. 2a). The positive LR was 4.60 (95% CI, 2.72–7.79), negative LR was 0.39 (95% CI, 0.29–0.52) (Table 2 and Additional file 1: Figure S1A) and the AUC was 0.86 (SE 0.02) (Fig. 3a). There was heterogeneity among the included studies.

**Table 2:** Pooled likelihood ratios of sputum Gram stain in community-acquired pneumonia

Microorganism	Positive LR (95% CI)	Negative LR (95% CI)
*S. pneumoniae*	4.60 (2.72–7.79)	0.39 (0.29–0.52)
*H. influenzae*	21.08 (8.32–53.40)	0.23 (0.13–0.41)
*S. aureus*	16.27 (2.48–106.86)	0.40 (0.30–0.56)
Gram-negative bacilli	37.49 (8.83–159.16)	0.45 (0.17–1.17)

Abbreviations: *LR* likelihood ratio, *CI* confidence interval

### *Haemophilus influenzae*

Eight studies were included in the analysis, comprising 3562 patients. Pooled sensitivity was 0.78 (95% CI, 0.72–0.84) and pooled specificity was 0.96 (95% CI, 0.94–0.97) (Fig. 2b and Additional file 1: Table S3). The positive LR was 21.08 (95% CI, 8.32–53.40), negative LR was 0.23 (95% CI, 0.13–0.41) (Table 2 and Additional file 1: Figure S1b) and AUC was 0.66 (SE: 0.10) (Fig. 3b). There was heterogeneity among the included studies.

### *Staphylococcus aureus*

Three studies that involved 1834 patients were included in this analysis. Pooled sensitivity was 0.72 (95% CI, 0.53–0.87) and pooled specificity was 0.97 (95% CI, 0.95–0.99) (Fig. 2c and Additional file 1: Table S4). The positive LR was 16.27 (95% CI, 2.48–106.86), negative LR was 0.40 (95% CI, 0.30–0.53) (Table 2 and Additional file 1: Figure S1C) and AUC was 0.78 (SE: 0.24) (Fig. 3c). There was heterogeneity among the studies.

### Gram-negative bacilli

Two studies were included comprising 1718 patients. Pooled sensitivity was 0.64 (95% CI, 0.49–0.77) and pooled specificity was 0.99 (95% CI, 0.97–0.99) (Fig. 2d and Additional file 1: Supplementary Table 5). The positive LR was 37.49 (95% CI, 8.83–159.16), negative LR was 0.45 (95% CI, 0.17–1.17) (Table 2 and Additional file 1: Figure S1D) and AUC was 0.94 (SE: 0.03) (Fig. 3d). There was heterogeneity among the studies included.

### Subgroup analyses

#### “Good quality” criteria

Nine studies of pneumococcal pneumonia used the “good quality” criteria for evaluating the sputum samples. Pooled sensitivity and pooled specificity were 0.59 and 0.93 respectively (Additional file 1: Table S6 and Figure S2A). Four studies for *H. influenzae* also used “good quality” criteria for their samples. The pooled sensitivity was 0.74 and the pooled specificity was 0.94 (Additional file 1: Table S7 and Figure S2B).

#### “Positive sputum Gram stain” criteria

Regarding the percentage of microorganisms seen in the sputum Gram stain (positive if presence of > 50% microorganisms of the same morphotype), we found a pooled sensitivity of 0.50 and pooled specificity of 0.93 for seven studies in patients with pneumococcal pneumonia (Additional file 1: Supplementary Table 8 and Additional file 1: Figure S3A). For *H. influenzae,* we found a pooled sensitivity of 0.78 and pooled specificity 0.97 (Additional file 1: Table S9 and Figure S3B).

#### Previous antibiotic therapy

In the studies that reported that patients with pneumococcal pneumonia did not receive antibiotics before the sample was taken, pooled sensitivity and specificity were both 0.78 (Additional file 1: Table S10 and Figure S4A). In the case of *H. influenzae*, we found a pooled sensitivity of 0.81 and a pooled specificity of 0.96 (Additional file 1: Table S11 and Figure S4B).

### Publication bias

**Fig. 4:**
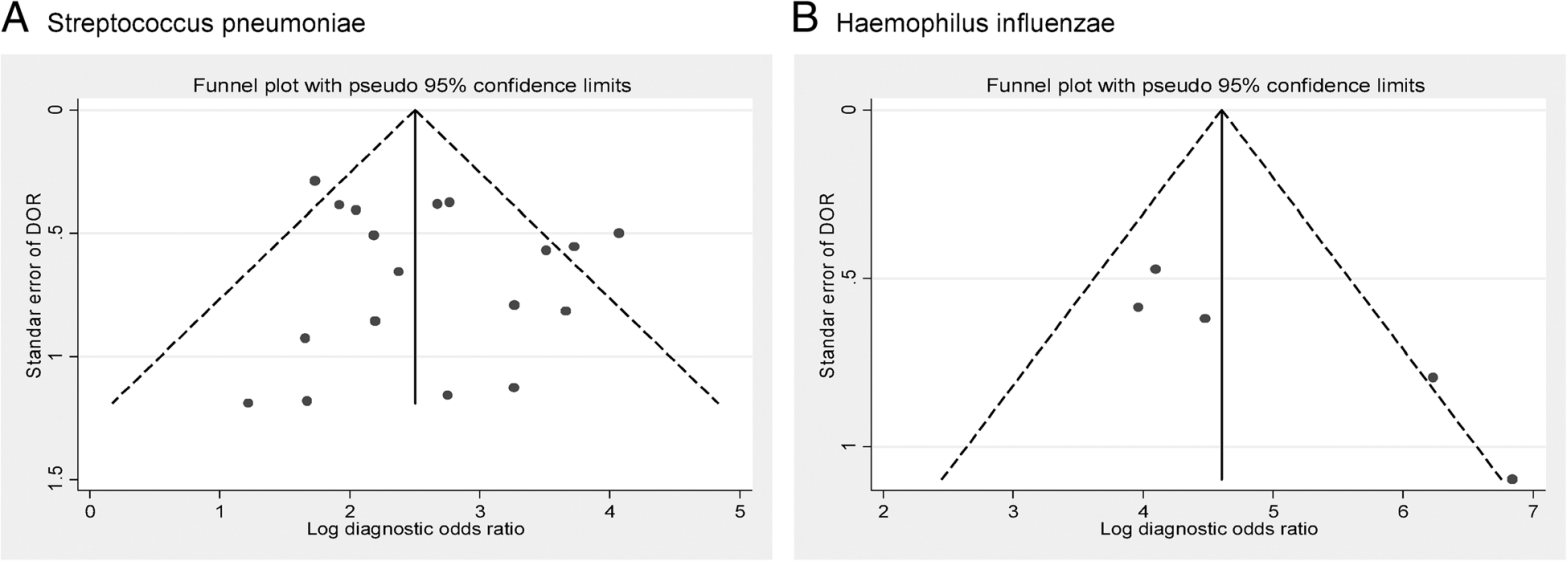
Funnel plot of studies regarding diagnostic accuracy of sputum Gram stain in community-acquired pneumonia . **a**. *Streptococcus pneumoniae*, **b**. *Haemophilus influenzae*

No publication or small-study bias was evident for the studies that evaluated pneumococcal pneumonia (Egger test *p* = 0.336), but the analysis of *H. influenzae* pneumonia indicated asymmetry and statistically significant evidence of publication or small-study bias (Egger test *p* = 0.031) (Fig. 4). For other causative pathogens of CAP, publication bias could not be analyzed due to the low number of studies.

## Discussion

This study evaluates the usefulness of sputum Gram stain to identify the causative pathogens in patients with CAP. The results of our meta-analysis demonstrated that the test is highly specific to identify *S. pneumoniae*, *H. influenzae*, *S. aureus* and Gram-negative bacilli infection. One of our most interesting findings is that the false-negative proportion for sputum Gram stain test ranges from 44% for *S. pneumoniae* to 22% for *H. influenzae*. This result suggests that stopping antimicrobials after a negative sputum Gram stain test result in patients may not be appropriate. Failure to detect these causative pathogens on sputum Gram stain does not conclusively indicate their absence. Similarly, none of the pooled negative LR achieved the result lower than 0.1, which is regarded as strong evidence to reliably exclude diagnoses. Thus, a negative sputum Gram stain produce a minor change in the probability of the etiologic diagnosis of CAP. However, a positive sputum Gram stain test result all but confirms the causative pathogen of CAP (with specificities ranging from 87% for *S. pneumoniae* to 99% for Gram-negative bacilli). These results suggest that, in patients with sputum production, a positive sputum Gram stain can lead to appropriate initial antibiotic selection. In this regard, the pooled positive LR was high (above 4 for *S. pneumoniae* but higher than 10 for *H. influenzae*, *S. aureus* and Gram-negative bacilli). It has been suggested that LR values higher than 10 provides strong evidence to rule in diagnoses in most circumstances.

The use of several microbiologic tests at the same time (extensive diagnostic testing) to determine the causative pathogen of CAP is controversial. There are several arguments in its favor: for instance, its results are likely to change antimicrobial management, it can have epidemiologic consequences such as *Legionella* infection or methicillin-resistant *S. aureus*, and it may provide the frequencies of etiology and resistant microorganisms [2]. However, the main disadvantage of these tests is their cost. The sputum Gram stain offers most of the advantages of diagnostic tests in CAP and, in addition, it is a rapid, simple and low-cost method. Similarly, the sputum Gram stain can identify causative pathogens such as *S. aureus* or Gram-negative bacilli that are missed by other tests, thus increasing the likelihood of appropriate antimicrobial use and, consequently, decreasing poor outcomes.

Only one meta-analysis that evaluated patients with pneumococcal pneumonia has investigated the accuracy of the sputum Gram stain in CAP. Reed et al. [9] concluded that the Gram stain might yield erroneous results, as its sensitivity and specificity differ substantially in diverse settings. In agreement with the findings reproduced in our meta-analysis regarding pneumococcal pneumonia, Reed et al. [9] reported that the studies included in their meta-analysis used different reference standards for the sputum Gram stain and different definitions of positivity, and that the preparation and interpretation was performed by personal from different services in each study. These methodological inconsistencies may explain the variations in the sensitivity and the specificity of the sputum Gram stain. Moreover, we also found heterogeneity in the methodology and the sensitivity and specificity among studies evaluating other causative pathogens such as *H. influenzae*, *S. aureus* and Gram-negative bacilli. On the other hand, although the present study evaluated CAP patients, two meta-analysis have assessed the role of bacteriological information in improving the clinical diagnosis of ventilator-associated pneumonia (VAP) with contradictory conclusions [32, 33]. However, Rea-Neto et al. [33] found that sputum Gram stain could be useful in early therapeutic decisions in VAP patients but may be influenced by prior antimicrobial use and the causative pathogen.

To overcome these drawbacks, we performed subgroup analyses including studies that reported “good quality” sputum, “positive sputum Gram stain”, and in which patients had not received prior antimicrobial therapy. The sensitivity and the specificity of the sputum Gram stain in the subgroups were comparable to those reported in the overall analyses. It has been suggested that the limited value of sputum Gram stain test is due to the difficulty of obtaining samples, or more precisely good quality samples [21, 22]. Studies have reported that nearly half of patients with CAP have sputum production. Moreover, although the definition of good quality is not consistent among studies, reports of good-quality sputum frequencies range from 14 to 71% [6, 34–37]. In our meta-analysis, we analyzed a subgroup of patients with “good quality” sputum, defined as > 25 neutrophils in a × 100 microscopic field and few squamous epithelial cells (< 10 in a × 100 microscopic field) (we stress that some of the studies included in the meta-analysis used different definitions of good quality sputum). The results for sensitivity and specificity in the subgroups were similar to the results of the overall analysis. Importantly, the studies did not report the usefulness of diagnostic tests when poor quality sputum was obtained. Similarly, the criteria used to define significance (the percentage of microorganisms seen in the Gram stain) varied between studies, but we did not detect major differences in these subgroup analyses compared with the overall results. Finally, we detected a higher specificity of sputum Gram stain for determining the etiology of CAP when patients had not previously received antibiotic therapy.

Other tests also use respiratory samples for the diagnosis of causative pathogens of CAP. Although polymerase chain reaction (PCR) testing performed in sputum can increased microbiological yield, it has been suggested that PCR is an unreliable diagnostic tool for pneumococcal pneumonia because it cannot differentiate between colonization and lower respiratory tract infection. In addition, other studies have found that PCR did not show significant increase in either sensitivity or specificity in pneumococcal pneumonia, even when quantification was included [38–41]. In this regard, Stralin et al. [42] and Kakiuchi et al. [43] obtained high sensitivity and specificity using PCR assays for *S. pneumoniae* applied to sputum and nasopharyngeal aspirate samples in patients with CAP. Moreover, protected specimen brush and bronchoalveolar lavage quantitative cultures added nearly 30% more microbiological documentation for CAP compared to sputum cultures. However, importantly, Gram staining was indicative of the pathogen mostly in cases where *S. pneumoniae* was isolated [44]. Major limitations of application of these tests include cost and the need for specialized medical equipment. Conversely, it has been documented that the use of Gram staining not appear to modify health care costs of CAP. A study found that cost reduction was more influenced be price differences between targeted therapy and non-targeted therapy [45].

Only one study has examined the impact of the use of sputum Gram stain to guide therapeutic decision-making on prognosis in hospitalized patients with CAP. Fukuyama et al. (6) reported that pathogen-targeted treatment guided by sputum Gram stain had similar efficacy and less adverse events than empirical treatment. These results were similar to those found in a randomized prospective open study in which a pathogen-directed approach was compared with empirical treatment in patients with CAP [46]. The pathogen-directed approach used clinical presentation and the results of a Gram stain from sputum or pleural fluid, pneumococcal antigen detection in sputum or pleural fluid, and *L. pneumophila* serogroup 1 urinary antigen detection test to determine the antibiotic therapy. No significant differences were found between the two treatment groups in length of stay, 30-day mortality, or clinical failure. Side effects occurred more frequently in patients in the empirical broad spectrum antibiotic treatment group. These results suggest that a positive sputum Gram stain can lead to appropriate initial antimicrobial selection without altering clinical outcome in patients with CAP. This idea is supported by the high specificity of the test, as reported in this meta-analysis, which allows accurate identification of the causative pathogen. However, more investigation is needed regarding about the potential impact of sputum Gram stain in clinical practice, particularly in the context of antimicrobial stewardship programs.

The main strength of this study is that it is the first meta-analysis to evaluate the usefulness of sputum Gram stain for determining the etiology of CAP due to diverse causative pathogens. In addition, because of the heterogeneity of the studies we also performed subgroup analyses, which consistently reported high specificity for the sputum Gram stain test. However, it should be note that one of the major concerns in the sputum Gram stain is its low reliability. The interpretation of the test is subjective and it is possible that differs by readers. Several limitations in our analysis should be acknowledged. First, we restricted our search to manuscripts published in Spanish and English. Second, we were unable to contact all author groups to obtain all relevant data in order to clarify discrepancies or request unpublished data. Third, the results should be interpreted cautiously because of limitations inherent to observational studies and the heterogeneity among studies. In addition, the methodologies used in microbiological work-up were heterogeneous. Fourth, a publication bias was detected for *H. influenzae* pneumonia, and this analysis could not be performed for *S. aureus* and Gram-negative bacilli pneumonia because of the low number of studies included. Finally, because there is no gold-standard for identifying the causative pathogen of CAP, the performance of sputum Gram stain may have been underestimated.

## Conclusion

Sputum Gram stain test is sensitive and highly specific for identifying causative pathogens in adult patients with CAP. Studies evaluating the impact of the use of sputum Gram stain in treatment decision-making on outcomes in hospitalized patients with CAP are now needed.

## Additional file

Additional file 1: Supplementary material. Usefulness of Sputum Gram Stain for Etiologic Diagnosis in Community-Acquired Pneumonia: A Systematic Review and Meta-Analysis. This file contains **Figure S1** (LR analysis); **Figure S2A**, Subgroup analysis of good quality sputum Gram stain in *S. pneumoniae*; **Figure S2B**, Subgroup analysis of good quality sputum Gram stain in *H. influenzae*; **Figure S3A**. Subgroup analysis of positive sputum Gram stain in *S. pneumoniae*; **Figure S3B**, Subgroup analysis of positive sputum Gram stain in *H. influenzae*; **Figure S4A**, Subgroup analysis of sputum Gram stain in previous antibiotics for *S. pneumoniae*; **Figure S4B**. Subgroup analysis of sputum Gram stain in previous antibiotics for *H. influenzae*; **Table S1**. QUADAS-2 Analysis; **Table S2**. Description of datasets included in the analysis of *S. pneumoniae*; **Table S3**. Description of datasets included in the analysis of *H. influenzae*; **Table S4**. Description of datasets included in the analysis of *S. aureus*; **Table S5**. Description of datasets included in the analysis of Gram-negative bacilli; **Table S6**. Description of datasets included in the subgroup analysis of good quality sputum Gram stain in *S. pneumoniae*; **Table S7**. Description of datasets included in the subgroup analysis of good quality sputum Gram stain in *H. influenzae*; **Table S8**. Description of datasets included in the subgroup analysis of positive sputum Gram stain in *S. pneumoniae*; **Table S9**. Description of datasets included in the subgroup analysis of positive sputum Gram stain in *H. influenzae*; **Table S10**. Description of datasets included in the subgroup analysis of sputum Gram stain in previous antibiotics for *S. pneumoniae*, and **Table S11**. Description of datasets included in the subgroup analysis of sputum Gram stain in previous antibiotics for *H. influenzae*. (DOCX 131 kb)

## Abbreviations

AUC: Area under the curve
CAP: Community-acquired pneumonia
CI: Confidence interval
LR: Likelihood ratios
ROC: Receiver operating characteristic curves
