# Supplementary material for: Usefulness of sputum gram stain for etiologic diagnosis in community-acquired pneumonia: a systematic review and meta-analysis
Source: BMC Infect Dis. 2019 May 10;19:403. doi: 10.1186/s12879-019-4048-6 (PMC6509769; doi:10.1186/s12879-019-4048-6)
Supplement: Supplementary file 1 — Supplementary material. Usefulness of Sputum Gram Stain for Etiologic Diagnosis in Community-Acquired Pneumonia: A Systematic Review and Meta-Analysis. This file contains Figure S1 (LR analysis); Figure S2A, Subgroup analysis of good quality sputum Gram stain in S. pneumoniae; Figure S2B, Subgroup analysis of good quality sputum Gram stain in H. influenzae; Figure S3A. Subgroup analysis of positive sputum Gram stain in S. pneumoniae; Figure S3B, Subgroup analysis of positive sputum Gram stain in H. influenzae; Figure S4A, Subgroup analysis of sputum Gram stain in previous antibiotics for S. pneumoniae; Figure S4B. Subgroup analysis of sputum Gram stain in previous antibiotics for H. influenzae; Table S1. QUADAS-2 Analysis; Table S2. Description of datasets included in the analysis of S. pneumoniae; Table S3. Description of datasets included in the analysis of H. influenzae; Table S4. Description of datasets included in the analysis of S. aureus; Table S5. Description of datasets included in the analysis of Gram-negative bacilli; Table S6. Description of datasets included in the subgroup analysis of good quality sputum Gram stain in S. pneumoniae; Table S7. Description of datasets included in the subgroup analysis of good quality sputum Gram stain in H. influenzae; Table S8. Description of datasets included in the subgroup analysis of positive sputum Gram stain in S. pneumoniae; Table S9. Description of datasets included in the subgroup analysis of positive sputum Gram stain in H. influenzae; Table S10. Description of datasets included in the subgroup analysis of sputum Gram stain in previous antibiotics for S. pneumoniae, and Table S11. Description of datasets included in the subgroup analysis of sputum Gram stain in previous antibiotics for H. influenzae. (DOCX 131 kb) [file 12879_2019_4048_MOESM1_ESM.docx]

**Usefulness of Sputum Gram Stain for Etiologic Diagnosis in Community-Acquired Pneumonia: A Systematic Review and Meta-Analysis**

Gaspar Del Rio-Pertuz, Juan F. Gutiérrez , Abel J. Triana, Jorge L. Molinares, Andrea B. Robledo-Solano, José L. Meza, Orlando M. Ariza-Bolívar, Jorge Acosta-Reyes, Argenis Garavito, Diego Viasus, Jordi Carratalà

BMC Infectious Diseases

Supplementary Figure 1

1. *Streptococcus pneumoniae*

1. *Haemophilus influenzae*

1. Staphylococcus aureus

1. Gram negative bacilli

Supplementary Figure 2A. Subgroup analysis of good quality sputum Gram stain in *S. pneumoniae*.

Supplementary Figure 2B. Subgroup analysis of good quality sputum Gram stain in *H. influenzae*.

Supplementary Figure 3A. Subgroup analysis of positive sputum Gram stain in *S.pneumoniae.*

Supplementary Figure 3B. Subgroup analysis of positive sputum Gram stain in *H. influenzae*

Supplementary Figure 4A. Subgroup analysis of sputum Gram stain in previous antibiotics for *S. pneumoniae*

Supplementary Figure 4B. Subgroup analysis of sputum Gram stain in previous antibiotics for *H. influenza*

Abbreviations: LR, Low risk; HR, High risk; ?, unclear risk

| Supplementary Table 1. QUADAS-2 Analysis | | | | | | | |
| --- | --- | --- | --- | --- | --- | --- | --- |
| Study | **Risk of Bias** | | | | **Applicability Concerns** | | |
|  | **Patient Selection** | **Index Test** | **Reference standard** | **Flow and timing** | **Patient selection** | **Index test** | **Reference standards** |
| Xiaoping, et al. | LR | HR | HR | HR | LR | HR | LR |
| Fukuyama, et al. | LR | LR | LR | HR | LR | LR | LR |
| Lim, et al. | LR | LR | LR | HR | LR | HR | LR |
| Anevlavis, et al. | HR | LR | LR | LR | LR | LR | LR |
| Rein, et al. | ? | LR | LR | LR | LR | LR | LR |
| Ewig, et al. | LR | LR | LR | LR | LR | LR | LR |
| Miyashita, et al. | LR | LR | LR | HR | LR | LR | LR |
| BTS. | HR | HR | ? | HR | LR | LR | LR |
| Musher, et al. | LR | HR | HR | HR | LR | LR | LR |
| Bohte, et al. | LR | ? | LR | HR | LR | LR | LR |
| Ferré, et al. | LR | ? | LR | HR | LR | LR | LR |
| Fine, et al. | LR | LR | LR | HR | LR | LR | LR |
| Belliveau, et al. | LR | LR | LR | LR | LR | LR | LR |
| Dans, et al. | LR | LR | LR | HR | LR | LR | LR |
| Lentino, et al. | LR | LR | LR | HR | LR | LR | LR |
| Rosón, et al. | LR | LR | LR | HR | LR | LR | LR |
| Merrill, et al. | HR | HR | LR | LR | HR | HR | LR |
| Gleckman, et al. | LR | LR | LR | LR | LR | LR | LR |
| Thorsteinsson, et al. | HR | HR | ? | LR | LR | LR | LR |
| Boerner, et al. | LR | LR | LR | HR | LR | LR | LR |

**Supplementary Table 2. Description of datasets included in the analysis of *S. pneumoniae***

| Author | Year | Journal | Patients | Definition of positive gram stain | Definition of good quality sample | Number of samples | | Microorganism | Sensitivity  (CI 95%) | Specificity  (CI 95%) |
| --- | --- | --- | --- | --- | --- | --- | --- | --- | --- | --- |
| Merrill, et al. | 1973 | N Engl J Med | 27 | Unknown | Unknown | | 53 | *S. pneumonia* | 96 (81-100) | 12 (2-30) |
|  |  |  |  |  |  |  | 30 |  | 43 (18-71) | 88 (62-98) |
|  |  |  |  |  |  | |  |  |  |  |
| Thorsteinsson, et al. | 1975 | JAMA | 16 | Unknown | Unknown | | 16 | *S. pneumoniae* | 100 (75-100) | 67 (9-99) |
|  |  |  |  |  |  | |  |  | 100 (75-100) | 67 (9-99) |
|  |  |  |  |  |  | |  |  | 100 (74-100) | 67 (9-99) |
| Rein, et al. | 1978 | JAMA | 42 | >50% of the same morphotype or >10/oif of the same morphotype | At least 10 PMN leukocytes | | 42 | *S. pneumoniae* | 62 (42-79) | 85 (55-98) |
| Boerner, et al. | 1982 | JAMA | 89 | >50% of the same morphotype | PMN leukocytes in excess of epithelial cell | | 76 | *S. pneumoniae* | 94 (79-99) | 64 (49-78) |
| Dans, et al. | 1984 | Arch Intern Med | 241 | Unknown | Used a ratio of PMN leukocytes:epithelial cells | | 154 | *S. pneumoniae* | 52 (40-65) | 88 (79-94) |
|  |  |  |  |  |  | | 147 |  | 63 (48-76) | 80 (71-88) |
| BTS. | 1987 | QJM | 511 | >50%of the same morphotype | Unknown | | 404 | *S. pneumoniae* | 15 (9-22) | 98 (96-99) |
| Lentino, et al. | 1987 | J ClinMicrobiol | 249 | >50%of the same morphotype | >25 leukocytes and <10 epithelial cells | | 40 | *S. pneumoniae* | 56 (31-78) | 95 (77-100) |
| Gleckman, et al. | 1988 | J ClinMicrobiol | 144 | >10/oif of the same morphotype | >25 leukocytes and <10 epithelial cells | | 59 | *S. pneumoniae* | 69 (52-84) | 83 (61-95) |
| Xiaoping, et al. | 1988 | MedMicrobiol  Inmmunol | 105 | >10/oif of the same morphotype | >25 leukocytes and <10 epithelial cells | | 95 | *S. pneumoniae* | 88 (62-98) | 85 (75-92) |
| Lim, et al. | 1989 | Med J Aust | 106 | >50% of the same morphotype or >10/oif of the same morphotype | Unknown | | 40 | *S. pneumoniae* | 68 (48-84) | 100 (74-100) |
| Fine, et al. | 1991 | J Gen InternMed | 170 | >50%of the same morphotype | >25 leukocytes and <10epithelial cells | | 36 | *S. pneumoniae* | 86 (42-100) | 72 (53-87) |
| Bohte, et al. | 1996 | Eur J ClinMicrobiol Infect Dis | 268 | Unknown | >25 leukocytes and <10 epithelial cells | | 268 | *S. pneumoniae* | 65 (53-75) | 76 (69-82) |
|  |  |  |  |  |  | |  |  |  |  |
| Rosón, et al. | 2000 | Clin Infect Dis | 533 | >75%of the same morphotype | >25 leukocytes and <10 epithelial cells | | 210 | *S. pneumoniae* | 57 (46-68) | 97 (92-99) |
| Ewig, et al. | 2001 | Chest | 116 | Predominant morphotype | >25 leukocytes and <10 epithelial cells | | 23 | *S. pneumoniae* | 50 (7-93) | 84 (60-97) |
| Musher, et al. | 2004 | ClinInfectDis | 105 | Predominant morphotype | 10 leukocytes for each epithelial cell | | 105 | *S. pneumoniae* | 31 (23-41) | NA |
| Miyashita, et al. | 2008 | MedSciMonit | 347 | Predominant morphotype | >25 leukocytes and <10 epithelial cells | | 124 | *S. pneumoniae* | 68 (52-82) | 94 (86-98) |
| Anevlavis, et al. | 2009 | J Infect | 1390 | >50% of the same morphotype | Sum of two scores >1 | | 178 | *S. pneumoniae* | 82 (72-89) | 93 (85-97) |
| Ferré, et al. | 2011 | Emergencias | 608 | >75%of the same morphotype | >25 leukocytes and <10 epithelial cells | | 294 | *S. pneumoniae* | 47 (39-56) | 94 (89-97) |
| Fukuyama, et al. | 2014 | BMC Infect Dis | 328 | >10/oif of the same morphotype | <10/oif epithelial cells and >10/oif PMN | | 218 | *S. Pneumoniae* | 66 (52-78) | 89 (84-94) |

Abbreviations: oif,oil immersion field; PNM, polymorphonuclear

**Supplementary Table 3. Description of datasets included in the analysis of *H. influenzae***

| Author | Year | Journal | Patients | Definition of positive gram stain | Definition of good quality sample | | Number of samples | Microorganism | | Sensitivity  (CI 95%) | | Specificity  (CI 95%) | |
| --- | --- | --- | --- | --- | --- | --- | --- | --- | --- | --- | --- | --- | --- |
| Thorsteinsson, et al. | 1975 | JAMA | 16 | Unknown | Unknown | 16 | | | *H. Influenzae* | | 100 (40-100) | | 100 (74-100) |
| Belliveau, et al. | 1993 | Pharmacotherapy | 224 | Unknown | Number of neuthrophils and epithelial cells | 319 | | | *H. influenzae* | | 88 (68-97) | | 99 (97-100) |
| Rosón, et al. | 2000 | Clin Infect Dis | 533 | >75%of the same morphotype | >25 leukocytes and <10 epithelial cells | 210 | | | *H. influenzae* | | 82 (65-93) | | 100 (97-100) |
| Ewig, et al. | 2001 | Chest | 116 | Predominant morphotype | >25 leukocytes and <10 epithelial cells | 23 | | | *H. Influenzae* | | 0 (0-60) | | 0 (0-18) |
| Miyashita, et al. | 2008 | MedSciMonit | 347 | Predominant morphotype | >25 leukocytes and <10 epithelial cells | 124 | | | *H. influenzae* | | 78 (52-94) | | 100 (97-100) |
| Anevlavis, et al. | 2009 | J Infect | 1390 | >50% of the same morphotype | Sum of two scores >1 | 178 | | | *H. influenzae* | | 79 (59-92) | | 96 (91-99) |
| Ferré, et al. | 2011 | Emergencias | 608 | >75%of the same morphotype | >25 leukocytes and <10 epithelial cells | 169 | | | *H. influenzae* | | 73 (52-88) | | 95 (90-98) |
| Fukuyama, et al. | 2014 | BMC Infect Dis | 328 | >10/oif of the same morphotype | <10/oif epithelial cells and >10/oif PMN | 218 | | | *H. influenzae* | | 78 (64-88) | | 95 (90-97) |

Abbreviations:oif,oil immersion field; PNM, polymorphonuclear

**Supplementary Table 4. Description of datasets included in the analysis of *S. aureus***

| Author | Year | Journal | Patients | Definition of positive gram stain | Definition of  good quality sample | Number of samples | | Microorganism | Sensitivity  (CI 95%) | | | Specificity  (CI 95%) |  |
| --- | --- | --- | --- | --- | --- | --- | --- | --- | --- | --- | --- | --- | --- |
| Ewig, et al. | 2001 | Chest | 116 | Predominant morphotype | >25 leukocytes and <10 epithelial cells | 23 | *S. aureus* | | | 50 (1-99) | 81 (58-95) | | |
| Anevlavis, et al. | 2009 | J Infect | 1390 | >50% of the same morphotype | Sum of two scores >1 | 178 | *S. aureus* | | | 76 (55-91) | 96 (92-99) | | |
| Fukuyama, et al. | 2014 | BMC Infect Dis | 328 | >10/oif of the same morphotype | <10/oif epithelial cells and >10/oif PMN | 218 | *S. aureus* | | | 50 (1-99) | 100 (98-100) | | |

Abbreviations: oif, oil immersion field; PNM, polymorphonuclear

**Supplementary Table 5. Description of datasets included in the analysis of Gram negative bacilli**

| Author | Year | Journal | Patients | Definition of positive gram stain | Definition of good quality sample | | Number of samples | Microorganism | Sensitivity  (CI 95%) | Specificity  (CI 95%) |
| --- | --- | --- | --- | --- | --- | --- | --- | --- | --- | --- |
| Anevlavis, et al. | 2009 | J Infect | 1390 | >50% of the same morphotype | Sum of two scores >1 | 178 | | Gram negative | 78 (60-91) | 95 (90-98) |
| Fukuyama, et al. | 2014 | BMC Infect Dis | 328 | >10/oif of the same morphotype | <10/oif epithelial cells and >10/oif PMN | 218 | | *K. pneumoniae* | 50 (12-88) | 100 (97-100) |
|  |  |  |  |  |  |  | | *P. aeruginosa* | 22 (3-60) | 100 (98-100) |

Abbreviations:oif,oil immersion field; PNM, polymorphonuclear

**Supplementary Table 6. Description of datasets included in the subgroup analysis of good quality sputum Gram stain in *S. pneumoniae*.**

| Author | Year | Journal | Patients | Definition of positive gram stain | Definition of good quality sample | Number of samples | Microorganism | Sensitivity  (CI 95%) | Specificity  (CI 95%) |
| --- | --- | --- | --- | --- | --- | --- | --- | --- | --- |
| Lentino, et al. | 1987 | J ClinMicrobiol | 249 | >50% of the same morphotype | >25 leukocytes and <10 epithelial cells | 40 | *S. pneumoniae* | 56 (31-78) | 95 (77-100) |
| Gleckman, et al. | 1988 | J ClinMicrobiol | 144 | >10/oif of the same morphotype | >25 leukocytes and <10 epithelial cells | 59 | *S. pneumoniae* | 69 (52-84) | 83 (61-95) |
| Xiaoping, et al. | 1988 | Med MicrobiolInmunol | 105 | >10/oif of the same morphotype | >25 leukocytes and <10 epithelial cells | 95 | *S. pneumoniae* | 88 (62-98) | 85 (75-92) |
| Fine, et al. | 1991 | J Gen Interm Med | 170 | >50%of the same morphotype | >25 leukocytes and <10epithelial cells | 36 | *S. pneumoniae* | 86 (42-100) | 72 (53-87) |
| Bohte, et al. | 1996 | Eur J ClinMicrobiol Infect Dis | 268 | Unknown | >25 leukocytes and <10 epithelial cells | 268 | *S. pneumoniae* | 65 (53-75) | 76 (69-82) |
| Rosón, et al.[27] | 2000 | Clin Infect Dis | 533 | >75%of the same morphotype | >25 leukocytes and <10 epithelial cells | 210 | *S. pneumoniae* | 57 (46-68) | 97 (92-99) |
| Ewig, et al. | 2001 | Chest | 166 | Predominant morphotype | >25 leukocytes and <10 epithelial cells | 23 | *S. pneumoniae* | 50 (7-93) | 84 (60-97) |
| Miyashita, et al. | 2008 | Med Sci Monit | 347 | Predominant morphotype | >25 leukocytes and <10 epithelial cells | 124 | *S. pneumoniae* | 68 (52-82) | 94 (86-98) |
| Ferré, et al. | 2011 | Emergencias | 608 | >75%of the same morphotype | >25 leukocytes and <10 epithelial cells | 294 | *S. pneumoniae* | 47 (39-56) | 94 (89-97) |

Abbreviations:oif,oil immersion field; PNM, polymorphonuclear

**Supplementary Table 7. Description of datasets included in the subgroup analysis of good quality sputum Gram stain in *H.influenzae*.**

| Author | Year | Journal | Patients | Definition of positive gram stain | Definition of good quality sample | Number of samples | | Microorganism | Sensitivity  (CI 95%) | | Specificity  (CI 95%) |
| --- | --- | --- | --- | --- | --- | --- | --- | --- | --- | --- | --- |
| Rosón, et al. | 2000 | Clin Infect Dis | 533 | >75%of the same morphotype | >25 leukocytes and <10 epithelial cells | | 210 | *H. Influenzae* | 82 (65-93) | 100 (97-100) | |
| Ewig, et al. | 2001 | Chest | 116 | Predominant morphotype | >25 leukocytes and <10 epithelial cells | | 23 | *H. Influenzae* | 0 (0-60) | 0 (0-18) | |
| Miyashita, et al. | 2008 | Med SciMonit | 347 | Predominant morphotype | >25 leukocytes and <10 epithelial cells | | 124 | *H. Influenzae* | 78 (52-94) | 100 (97-100) | |
| Ferré, et al. | 2011 | Emergencias | 608 | >75%of the same morphotype | >25 leukocytes and <10 epithelial cells | | 169 | *H. Influenzae* | 73 (52-88) | 95 (90-98) | |

Abbreviations:oif,oil immersion field; PNM, polymorphonuclear

**Supplementary Table 8. Description of datasets included in the subgroup analysis of positive sputum Gram stain in *S.pneumoniae***

| Author | Year | Journal | Patients | Definition of positive gram stain | Definition of good quality sample | Number of samples | Microorganism | Sensitivity (CI 95%) | Specificity (CI 95%) |
| --- | --- | --- | --- | --- | --- | --- | --- | --- | --- |
| Boerner, et al. | 1982 | JAMA | 89 | >50% of the same morphotype | PMN leukocytes in excess of epithelial cell | 76 | *S. pneumoniae* | 94 (79 -99) | 64 (49 -78) |
| BTS. | 1987 | QJM | 511 | >50%of the same morphotype | Unknown | 404 | *S. pneumoniae* | 15 (9-22) | 98 (96-99) |
| Lentino, et al. | 1987 | J ClinMicrobiol | 249 | >50% of the same morphotype | >25 leukocytes and <10 epithelial cells | 40 | *S. pneumoniae* | 56 (31-78) | 95 (77-100) |
| Fine, et al. | 1991 | J Gen Interm Med | 170 | >50%of the same morphotype | >25 leukocytes and <10epithelial cells | 36 | *S. pneumoniae* | 86 (42-100) | 72 (53-87) |
| Rosón, et al. | 2000 | Clin Infect Dis | 533 | >75%of the same morphotype | >25 leukocytes and <10 epithelial cells | 210 | *S. pneumoniae* | 57 (46-68) | 97 (92-99) |
| Anevlavis, et al. | 2009 | J Infect | 1390 | >50% of the same morphotype | Sum of two scores >1 | 178 | *S. pneumoniae* | 82 (72-89) | 93 (85-97) |
| Ferré, et al. | 2011 | Emergencias | 608 | >75%of the same morphotype | >25 leukocytes and <10 epithelial cells | 294 | *S. pneumoniae* | 47 (39-56) | 94 (89-97) |

Abbreviations:oif,oil immersion field; PNM, polymorphonuclear

**Supplementary Table 9. Description of datasets included in the subgroup analysis of positive sputum Gram stain in *H.influenzae***

| Author | Year | Journal | Patients | Definition of positive gram stain | Definition of good quality sample | Number of samples | Microorganism | Sensitivity (CI 95%) | Specificity (CI 95%) |
| --- | --- | --- | --- | --- | --- | --- | --- | --- | --- |
| Rosón, et al. | 2000 | Clin Infect Dis | 533 | >75%of the same morphotype | >25 leukocytes and <10 epithelial cells | 210 | *H. Influenzae* | 82 (65-93) | 100 (97-100) |
| Anevlavis, et al. | 2009 | J Infect | 1390 | >50% of the same morphotype | Sum of two scores >1 | 178 | *H. Influenzae* | 79 (59-92) | 96 (91-99) |
| Ferré, et al. | 2011 | Emergencias | 608 | >75%of the same morphotype | >25 leukocytes and <10 epithelial cells | 169 | *H. Influenzae* | 73 (52-88) | 95 (90-98) |

Abbreviations:oif,oil immersion field; PNM, polymorphonuclear

**Supplementary Table 10. Description of datasets included in the subgroup analysis of sputum Gram stain in previous antibiotics for *S.pneumoniae***

| Author | Year | Journal | Patients | Definition of positive gram stain | Definition of good quality sample | Number of samples | Microorgansim | Sensitivity  (CI 95%) | Specificity (CI 95%) |
| --- | --- | --- | --- | --- | --- | --- | --- | --- | --- |
| Merrill, et al. | 1973 | NEJM | 27 | Unknown | Unknown | 53 | *S. pneumoniae* | 96 (81- 100) | 12 (2-30) |
|  |  |  |  |  |  | 30 |  | 43 (18-71) | 88 (62-98) |
| Thorsteinsson, et al. | 1975 | JAMA | 16 | Unknown | Unknown | 16 | *S. pneumoniae* | 100 (75-100) | 67 (9-99) |
|  |  |  |  |  |  |  |  | 100 (75-100) | 67 (9-99) |
|  |  |  |  |  |  |  |  | 100 (74-100) | 67 (9-99) |
| Rein, et al. | 1978 | JAMA | 42 | >50% of the same morphotypeor >10/oif of the same morphotype | At least 10 PMN leukocytes | 42 | *S. pneumoniae* | 62 (42-79) | 85 (55-98) |
| Gleckman, et al. | 1988 | J ClinMicrobiol | 144 | >10/oif of the same morphotype | >25 leukocytes and <10 epithelial cells | 59 | *S. pneumoniae* | 69 (52-84) | 83 (61-95) |
| Lim, et al. | 1989 | Med J Aust | 106 | >50% of the same morphotype or >10/oif of the same morphotype | Unknown | 40 | *S. pneumoniae* | 68 (48-84) | 100 (74-100) |
| Anevlavis, et al. | 2009 | J Infect | 1390 | >50% of the same morphotype | Sum of two scores >1 | 178 | *S. pneumoniae* | 82 (72-89) | 93 (85-97) |

Abbreviations:oif,oil immersion field; PNM, polymorphonuclear

**Supplementary Table 11. Description of datasets included in the subgroup analysis of sputum Gram stain in previous antibiotics for *H.influenzae***

| Author | Year | Journal | Patients | Definition of positive gram stain | Definition of good quality sample | Number of samples | Microorganism | Sensitivity (CI 95%) | Specificity (CI 95%) |
| --- | --- | --- | --- | --- | --- | --- | --- | --- | --- |
| Thorsteinsson, et al. | 1975 | JAMA | 16 | Unknown | Unknown | 16 | *H Influenzae* | 100 (40-100) | 100 (74-100) |
| Anevlavis, et al. | 2009 | J Infect | 1390 | >50% of the same morphotype | Sum of two scores >1 | 178 | *H. influenzae* | 79 (59-92) | 96 (91-99) |

Abbreviations:oif,oil immersion field; PNM, polymorphonuclear
